# GRAIMatter: Guidelines and Resources for AI Model Access from TrusTEd Research environments (GRAIMatter).

**DOI:** 10.23889/ijpds.v7i3.2005

**Published:** 2022-08-25

**Authors:** Emily Jefferson, Christian Cole, Alba Crespi Boixader, Simon Rogers, Maeve Malone, Felix Ritchie, Jim Smith, Francesco Tava, Angela Daly, Jillian Beggs, Antony Chuter

**Affiliations:** 1 University of Dundee; 2 NHS Scotland; 3 University of West of England; 4 PPIE Co-I

## Objectives

To assess a range of tools and methods to support Trusted Research Environments (TREs) to assess output from AI methods for potentially identifiable information, investigate the legal and ethical implications and controls, and produce a set of guidelines and recommendations to support all TREs with export controls of AI algorithms.

## Approach

TREs provide secure facilities to analyse confidential personal data, with staff checking outputs for disclosure risk before publication. Artificial intelligence (AI) has high potential to improve the linking and analysis of population data, and TREs are well suited to supporting AI modelling. However, TRE governance focuses on classical statistical data analysis. The size and complexity of AI models presents significant challenges for the disclosure-checking process. Models may be susceptible to external hacking: complicated methods to reverse engineer the learning process to find out about the data used for training, with more potential to lead to re-identification than conventional statistical methods.

## Results

GRAIMatter is:

- Evaluating a range of tools to determine effectiveness for disclosure control
- Assessing the legal and ethical implications of TREs supporting AI development and identifying aspects of existing legal and regulatory frameworks requiring reform.
- Running 4 PPIE workshops to understand their priorities and beliefs around safeguarding and securing data
- Developing a set of recommendations including
  - suggested open-source toolsets for TREs to use to measure and reduce disclosure risk
  - descriptions of the technical and legal controls and policies TREs should implement across the 5 Safes to support AI algorithm disclosure control
  - training implications for both TRE staff and how they validate researchers

## Conclusion

Records can be linked successfully, but estimated performance of record linkage depends on the validation set used. Manually reviewed data contain noise and may underestimate performance, while national ID numbers may overestimate performance due to non-random patterns of missingness.

